# Application and integration of deep learning in tumour radiomics: bibliometrics and visualisation analysis

**DOI:** 10.3389/frai.2026.1779682

**Published:** 2026-07-29

**Authors:** Yi Huang, Xinli Liu, Ying Zhou

**Affiliations:** 1 College of Clinical Medicine, Jining Medical University, Jining, Shandong, China; 2 Department of Radiology, Jinan Fourth People's Hospital, Jinan, Shandong, China

**Keywords:** artificial intelligence, deep learning, magnetic resonance imaging, radiomics, visual analysis

## Abstract

**Introduction:**

The integration of deep learning with tumour radiomics represents a significant advance in precision oncology, offering a powerful alternative to traditional radiomics that relies on manually designed imaging features and is limited in capturing tumour complexity.

**Methods:**

To analyze integration strategies, trends, and future directions in this field, a bibliometric analysis was conducted using the Web of Science Core Collection, retrieving 4,915 relevant articles as of October 28, 2025. Data were examined with CiteSpace, VOSviewer, and bibliometrix, employing co-authorship, co-citation, keyword analysis, Latent Dirichlet Allocation topic modeling, and burst detection.

**Results:**

Annual publications rapidly increased from 253 in 2019 to 1,021 in 2024, with China and the United States as leading contributors and the U.S. serving as the central hub for international collaboration. Eighteen core topics were identified and grouped into four domains: tumour characterization and diagnosis, treatment response and prognosis, molecular profiling, and methodological innovation. The field has evolved from early texture analysis toward current focuses on multimodal fusion, molecular subtyping, and deep learning-driven predictive modeling.

**Discussion:**

This bibliometric analysis suggests a clear transition in oncological image analysis from descriptive morphology toward predictive and prognostic modeling, driven by the deep integration of deep learning with radiomics. Future efforts should prioritize multinational collaboration, methodological standardization, and prospective clinical validation to help bridge the gap toward clinical integration, while acknowledging that reproducibility and other validation challenges persist.

## 1 Introduction

The precise diagnosis, evaluation of efficacy, and prognostic prediction of tumours are key challenges in modern clinical practice. With medical imaging technology rapidly developing, imaging modalities, including computed tomography (CT), magnetic resonance imaging (MRI), and positron emission tomography (PET), have generated massive amounts of data rich in biological information (Distefano et al., 2024; Chen et al., 2025). Against this backdrop, radiomics has emerged. Its fundamental concept is to extract massive amounts of quantitative imaging features that are invisible to the human eye through high-throughput processing, transform medical images into high-dimensional data that can be analysed, and build models to noninvasively decode the heterogeneity within tumours and assist in clinical decision-making (Hsu et al., 2025). Traditional radiomics analysis workflows generally follow a “hand-designed feature” paradigm, including image acquisition and reconstruction, region of interest segmentation, feature extraction and screening, and model building. This paradigm has shown great potential in various tumour types (Jin et al., 2025); however, its limitations are becoming increasingly evident. First, extracting hand-designed features heavily depends on accurate image segmentation, a time-consuming process that is susceptible to inter-observer variability. Second, these predefined features may not fully capture the complex image patterns that are most relevant to tumour biological behaviour, leading to limited model generalisation ability (Kawahara et al., 2025).

Deep learning is a revolutionary technology in artificial intelligence (AI) that has significantly advanced in computer vision tasks owing to its powerful end-to-end feature learning and hierarchical representation capabilities. Unlike traditional methods that depend on pre-existing human knowledge, deep learning models such as convolutional neural networks (CNNs) can automatically learn and extract highly discriminative feature representations from raw images, thus potentially overcoming the dependence of traditional radiomics on manual design and uncovering deeper and more universal image biomarkers (Nguyen-Tat et al., 2025). Currently, the combination of deep learning and radiomics is evolving from “parallel” to “deep integration,” which can be observed at three levels:

Replacement and enhancement: Using deep learning models (such as U-Net) to achieve accurate and automated tumour segmentation, laying a reliable foundation for subsequent feature extraction; or directly using pre-trained CNN models as powerful feature extractors to generate “deep learning features” (Ajay et al., 2025; Nguyen-Tat et al., 2025).

Integration and complementarity: Features automatically extracted by deep learning are integrated with traditionally hand-designed radiomics features, combining the advantages of both to construct a more robust and comprehensive predictive model (Kawahara et al., 2025).

End-to-End Learning: Constructing an integrated deep learning framework directly from raw image input to the final clinical endpoint output (including survival prediction and genotyping), achieving true end-to-end modelling, minimising information loss, and human intervention (Hsu et al., 2025).

The aim of this study was to systematically review the innovative applications and deep integration strategies of deep learning technology in tumour radiomics. First, we outline the basic process and challenges of traditional radiomics and then delve into the methodological progress of deep learning in key aspects, including image segmentation, feature extraction, and model construction. The focus will be on the latest research dynamics and future prospects of deep learning and radiomics in multimodal data fusion, interpretability analysis, and clinical translation pathways to provide new ideas and directions for promoting the development of precision medicine.

## 2 Methods

### 2.1 Data download

There are multiple biomedical databases in scientometrics research, including the Web of Science (WOS), Scopus, PubMed, MEDLINE, and Embase. Integrating different databases can provide more information; nevertheless, a large amount of duplicate data exists across them. Combining data from different sources may introduce unnecessary confounding factors, influence data quality, and ultimately affect the experimental conclusions. Choosing a single, high-quality, authoritative database as the data source is a routine strategy in scientometric analysis. This represents the main trends and directions of the entire research field, minimises confounding factors, and ensures data quality. The WOS Core Database was selected as the data source for this informatics analysis owing to its comprehensiveness and authority (Guo et al., 2024).

**Figure 1:**
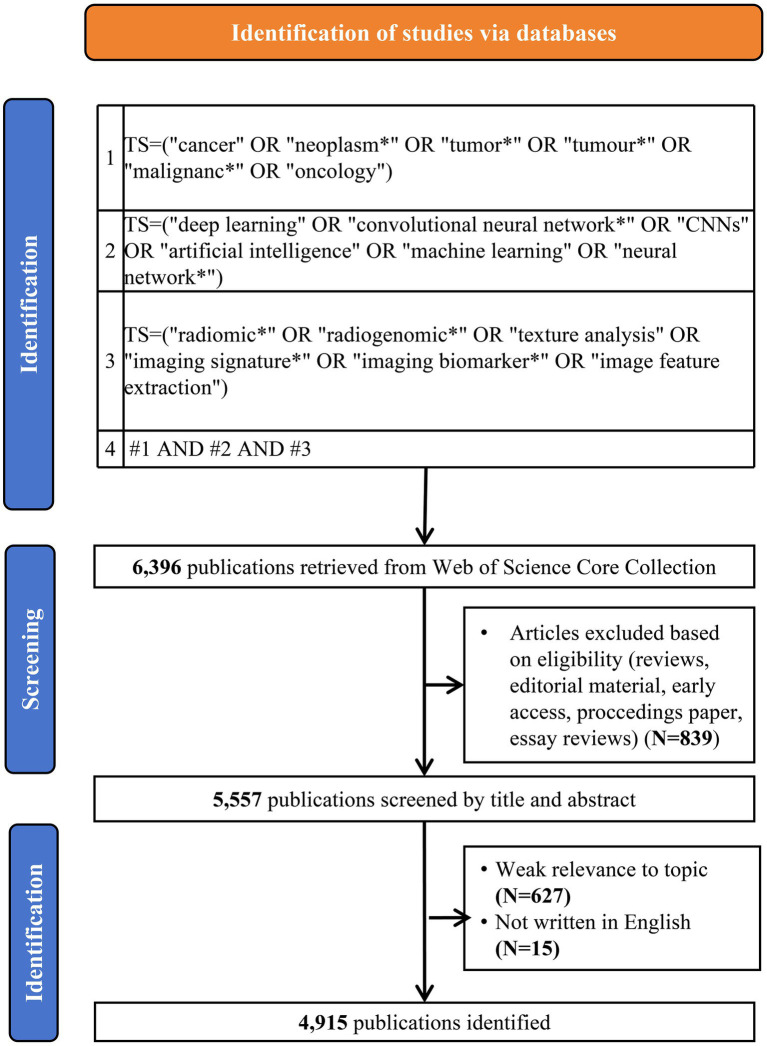
PRISMA flow diagram of the systematic literature search strategy and screening process.

On 28 October 2025, relevant keywords for “deep learning” and “radiomics” were compiled in the Web of Science Core Collection (WoSCC) database, and a comprehensive literature search was conducted with the language limited to English and the article type limited to Article. All data results were downloaded on the same day to avoid the impact of database updates. The file formats included plain text, BibTex, and tab-delimited files. Finally, 4,915 records were selected for the analysis. The detailed retrieval and screening processes are shown in Figure 1. The full search strategy is reported in Supplementary File 1.

### 2.2 Data analysis and visualisation

In this study, the retrieved data were imported into CiteSpace (version 6.3. R1), VOSviewer (version 1.6.20), the bibliometrix (version 4.3.0) package in R (version 4.4.0), KH Coder software (version 3b07d), and the online bibliometrics website^1^ to analyse published research and create visualisation maps.

VOSviewer conducts co-authorship, co-occurrence, and co-citation analyses on large-scale literature data to visualise knowledge graphs representing authors, journals, and other relevant information. It is widely used in bibliometric analyses (van Eck and Waltman, 2010; Qu et al., 2024). The different nodes represent authors, countries, institutions, journals, and keywords. The node size corresponds to the number of citations or references. The links between nodes represent cooperation and co-occurrence relationships. The colours of the nodes and lines represent different clusters, corresponding years, or average references. We also used CiteSpace (version 6.3. R1) to conduct a double graph overlay and cited journal clustering analysis to determine development dynamics and future trends in the research field (Chen, 2004, 2006). Furthermore, an international cooperation network was established between countries using an online scientometric platform.

Topic modelling is a natural language processing technique used to discover potential topics in public documents. Latent Dirichlet Allocation (LDA) is a popular topic modelling method that effectively handles large amounts of unstructured text (Stout et al., 2018). LDA operates by generating a term function vocabulary and analysing word frequency co-occurrences in documents. LDA calculates the relevance of an article to a topic after the vocabulary is built based on the word frequency in each document. The number of topics (K) was determined using various criteria, including pairwise cosine distance, Kullback–Leibler divergence, and model coherence (Cao et al., 2009; Arun et al., 2010; Mimno et al., 2011). As shown in Supplementary Figure S1, we compared several metrics for a range of K values, and K = 18 achieved the best balance across all indicators. The detailed evaluation results are reported in Supplementary File 2.

We used the theme significance ranking (TSR) score following the criteria proposed by AlSumait et al. (2009) to evaluate the quality of the topics. The TSR is a novel unsupervised analysis technique for probabilistic topic models (PTM). A 4-stage weighted combination decision strategy was used to evaluate the semantic significance of the PTM-inferred topics. This method combines information from multiple standards into a single evaluation index using a widely used weighted linear combination decision strategy. This technique allows topics to be ranked appropriately based on their true meanings. The ranking of topics depends on the distribution of the contextual words. Topics with higher TSR scores are better at capturing important topics and show meaningful class semantics.

Kleinberg (2003) proposed a Burst Detection algorithm, a method for identifying the period of abnormally frequent events in time-series data, which can also provide valuable insights for trend prediction. The early stage of a burst can be identified, and potential emerging trends can be warned about by analysing the frequency, duration, and intensity of historical bursts.

Raw data downloaded from the WoSCC were imported into Microsoft Excel 2024 for preliminary processing. We retained only the titles and abstracts of each article to form the original corpus. We established word-frequency thresholds, deleted general stop words, and defined specific stop words to ensure the reliability and effectiveness of the results. Python (version 3.10) was used for this process, and the scripts and code are available from the corresponding author upon request. LDA topic modelling and word clouds were conducted using KH Coder software and WordCloud. In the final stage, each topic was independently named by two researchers (Y.H. and X.L.), based on the 10 most prominent articles and 20 topic terms. All disagreements were first resolved through discussion; when consensus could not be reached, a third senior researcher (Y.Z.) was consulted to make the final decision. The detailed topic naming process is provided in Supplementary Sheet S9.

## 3 Results

### 3.1 Analysis of development trends and collaboration patterns

**Figure 2:**
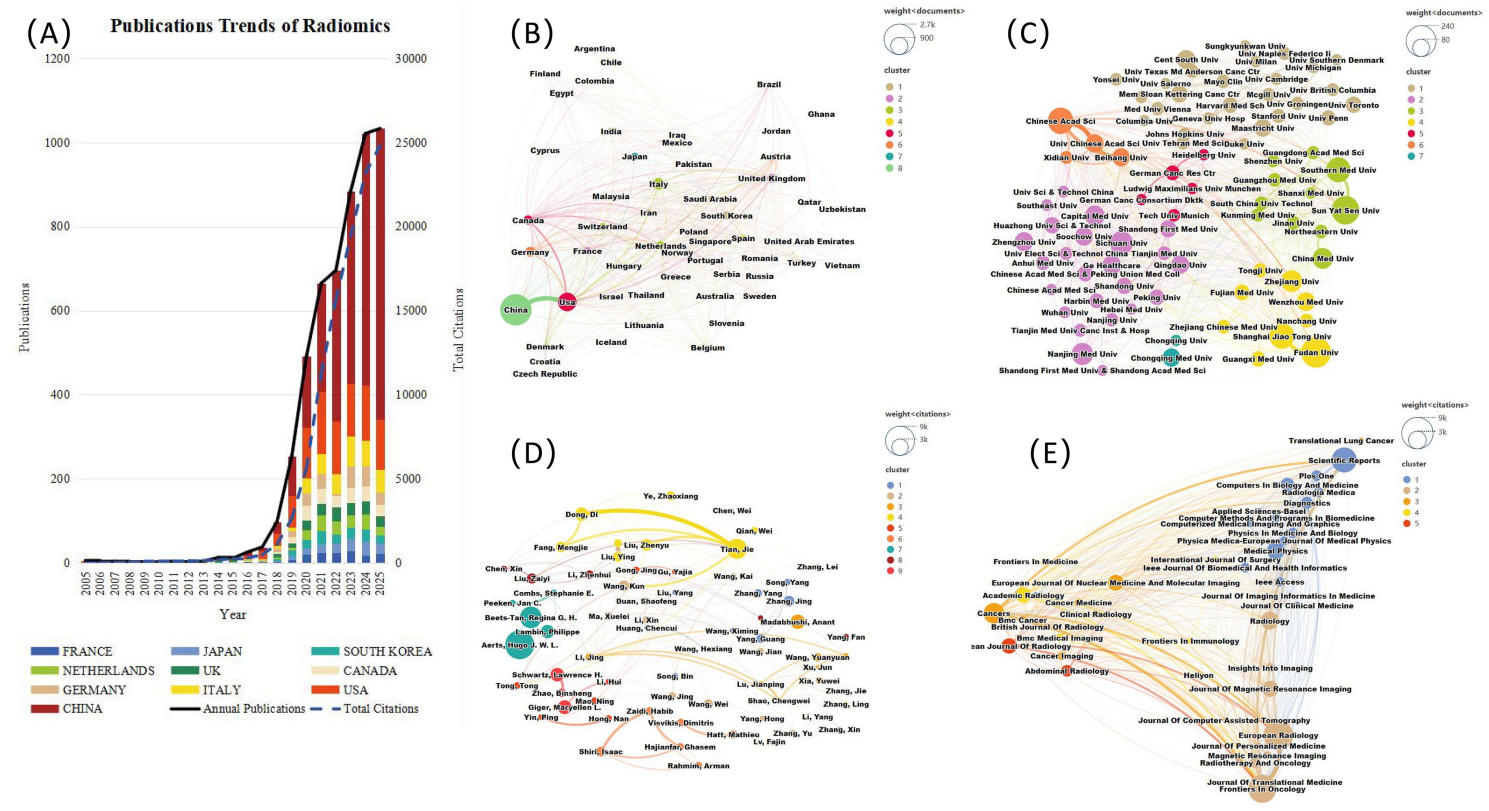
Annual publication trends and citation impact in the field.

Over the past two decades, the integration of deep learning with tumour radiomics has undergone a significant paradigm shift, particularly entering a phase of explosive growth since 2020 (Figure 2A). Bibliometric data reveal a clear evolution from modest beginnings to rapid expansion in annual output: the number of publications increased sharply from 253 in 2019 to 1,021 in 2024, marking the field’s entry into an unprecedentedly active period of prosperity and technological maturity.

In the evolving global research landscape, national contributions exhibit a notable dynamic shift, characterized by a bipolar structure led by China and the United States. In the early stages, the United States, with its first-mover advantage, was the primary driver in constructing the technical framework of the field. However, since 2020, China has achieved a critical leap in research output, surpassing the United States. By 2025, China’s annual publication volume reached 692 papers, accounting for more than two-thirds of the global total, demonstrating an absolute advantage in output scale. Alongside this dual-core leadership, the global research landscape shows multipolar collaboration. The United States maintains steady research strength, while other countries—such as Italy (54 papers in 2025), Germany, Canada, and the United Kingdom—serve as important supplementary forces, all showing steady growth in output (Table 1).

**Table 1:** Top 10 most productive countries by publication and citation counts.

Country	2005	2006	2007	2008	2009	2010	2011	2012	2013	2014	2015	2016	2017	2018	2019	2020	2021	2022	2023	2024	2025
France	0	0	0	0	0	0	0	0	0	0	1	0	0	3	7	21	24	24	29	19	23
Japan	0	0	0	0	0	0	0	0	0	1	0	0	0	2	10	15	21	24	28	32	25
South Korea	1	0	1	1	0	0	0	1	0	0	0	0	0	6	13	19	31	20	29	30	19
Netherlands	0	0	0	0	0	0	0	1	0	1	3	1	2	3	7	24	38	32	29	35	20
UK	0	0	0	0	0	0	1	0	0	3	3	3	1	8	10	22	17	33	20	32	26
Canada	0	0	0	0	0	2	1	0	1	0	0	2	1	4	15	36	25	28	20	36	27
Germany	1	0	0	0	0	0	0	0	1	0	0	2	3	9	14	30	36	26	33	47	28
Italy	0	0	1	0	0	0	1	0	0	1	0	1	4	7	10	35	48	68	61	60	54
USA	4	2	1	1	1	2	0	1	2	5	4	10	9	29	65	103	149	106	106	122	109
China	0	2	1	1	1	0	0	1	0	2	0	7	9	27	72	150	206	308	426	548	689
Annual publications	6	4	4	3	2	4	3	4	4	13	11	26	29	98	223	455	595	669	781	961	1,020
Total citations	417	589	342	297	215	330	189	187	335	557	1717	2,578	8,658	7,845	18,722	21,942	20,234	16,222	11,145	6,121	1,162

The international cooperation pattern of deep learning in the field of tumour radiomics was analysed in this study through co-authorship network analysis (Figure 2B). The results showed that a global research network was formed in this field; however, significant differences were observed in participation among countries. Regarding academic output, China (2,673 articles) and the United States (926 articles) were the two countries with the largest number of publications, far ahead of Germany, Italy, and Canada. Regarding international cooperation, the United States became the core hub of the network, with the highest overall link strength. Notably, China’s international cooperation intensity is relatively low compared to its huge publication volume. Conversely, the Netherlands shows a strong tendency towards international cooperation, which is a key support for its academic influence. Overall, developed countries in the United States and Europe dominate the international cooperation network, whereas high-yielding China can still develop in the depths of international cooperation.

The institutional co-authorship network of deep learning in the field of tumour radiomics was analysed in this study using VOSviewer, with a network comprising 70 research institutions (Figure 2C). The overall network was relatively tight, with Chinese institutions dominating publication volume and collaboration intensity. Institutions with the highest collaboration intensity include Sun Yat-sen University, Chinese Academy of Sciences, and Fudan University, which also rank high in publication volume, indicating that prolific institutions generally have extensive collaborative relationships. The network includes internationally renowned institutions, such as Harvard University and Massachusetts General Hospital Cancer Center; however, their link strength is relatively limited, suggesting that current collaborations in this field are primarily domestic. In summary, this research field has formed a collaborative network centred on major Chinese universities and research institutions.

Journal co-citation network analysis based on VOSviewer showed that deep learning forms a close academic exchange network in tumour radiomics (Figure 2D). This network was driven by several high-impact core journals, among which Cancers, European Radiology, Frontiers in Oncology, and Scientific Reports showed significant hub roles, possessing the highest citation frequency and the broadest connectivity. This indicates that these journals are key platforms for knowledge dissemination and integration in this field, connecting multiple sub-directions, including radiology, medical physics, computer science, and integrative medicine, and collectively forming the core knowledge foundation of this interdisciplinary field.

In terms of academic output, the distribution of publications in this field demonstrates the leading role of core journals. *Frontiers in Oncology* ranks first with 418 articles, reflecting its high activity in publishing relevant research. It is closely followed by *Cancers* (250 articles) and *European Radiology* (208 articles). Notably, according to the 2024 edition of JCR, seven of the top ten most productive journals are in the JCR Q1 category, indicating that high-output research in this field is mainly published on well-recognized, high-quality academic platforms. The stable contributions of journals such as *Scientific Reports*, *Academic Radiology*, and *Diagnostics* collectively form the major vehicles for research in this field, reflecting the central roles of clinical imaging, oncology, and medical physics in current research (Table 2).

**Table 2:** Top 10 journals.

Rank	Top productive journals				Top-cited journals				
	Journals	Productions	JCR	IF (2025)	Journals	Citations	Average citations	JCR	IF (2025)
1	Frontiers in Oncology	418	Q2	3.3	European Radiology	7,501	36.1	Q1	4.7
2	Cancers	250	Q2	4.4	Frontiers in Oncology	6,769	16.2	Q2	3.3
3	European Radiology	208	Q1	4.7	Scientific Reports	5,339	27.5	Q1	3.9
4	Scientific Reports	194	Q1	3.9	Cancer Research	4,185	963.0	Q1	16.6
5	Academic Radiology	136	Q1	3.9	Cancers	3,564	14.3	Q2	4.4
6	Diagnostics	117	Q1	3.3	Radiology	2,703	112.6	Q1	15.2
7	Medical Physics	103	Q1	3.2	Medical Physics	2,639	25.6	Q1	3.2
8	European Journal of Radiology	88	Q1	3.3	Journal of Magnetic Resonance Imaging	2,229	34.3	Q1	4.3
9	Abdominal Radiology	81	Q2	2.2	European Journal of Nuclear Medicine and Molecular Imaging	2086	36.6	Q1	7.6
10	BMC Medical Imaging	81	Q1	3.2	European Journal of Radiology	1987	22.6	Q1	3.3

Average Citations = Citations/Productions.

Citation count, as a key indicator of academic impact, reveals the core value of different journals in knowledge dissemination. Although *Frontiers in Oncology* leads in publication volume, *European Radiology* ranks first in total citations with 7,501 citations, making it the most academically influential journal in the field. More notably, some journals exhibit extremely high per-article impact. For example, *Cancer Research*, though not among the top ten in publication volume, has an average citation count of 963.0 per article, far exceeding other journals, demonstrating that the high-quality studies it publishes have profound penetration within the academic network. Similarly, *Radiology* (average citation 112.6) also reflects high research depth and authority. These data indicate that academic impact in this field depends not only on publication scale but more on the citation contribution of high-caliber research outcomes.

Integrating the dual dimensions of output and citation, the field has formed a multidisciplinary network based on clinical imaging diagnosis and centered on oncology research. Journals such as *European Radiology*, *Scientific Reports*, and *Medical Physics* perform well in both output and citation rankings, constituting the core backbone supporting the academic development of the field. The presence of highly cited journals such as the *European Journal of Nuclear Medicine and Molecular Imaging* and the *Journal of Magnetic Resonance Imaging* further demonstrates that molecular imaging and magnetic resonance technology are hotspot directions for producing high-quality outcomes in this field. Overall, the allocation of academic resources in this field is relatively concentrated, with leading journals playing an irreplaceable hub role in maintaining disciplinary activity and guiding research trends.

Based on the author’s co-citation network analysis using VOSviewer, the core academic community was observed in the field of “deep learning in tumour radiomics (Figure 2E). The analysis shows that Tian and Jie occupy a central position in this collaborative network, indicating that their research has a wide-ranging effect and connects multiple academic groups. Furthermore, authors such as Liu, Zhenyu, Dong, Di, Liu, Zaiyi, Wang, Kun, and Zhang, Jing also exhibit significant connection strength, collectively forming key hubs in the network. However, some authors have shown lower connection strengths, suggesting that their research directions are relatively independent. This network structure revealed a closed-knit collaborative group centred on Tian and Jie, comprising multiple high-influence scholars, outlining the mainstream academic collaboration pattern in this field.

### 3.2 Keyword analysis

**Figure 3:**
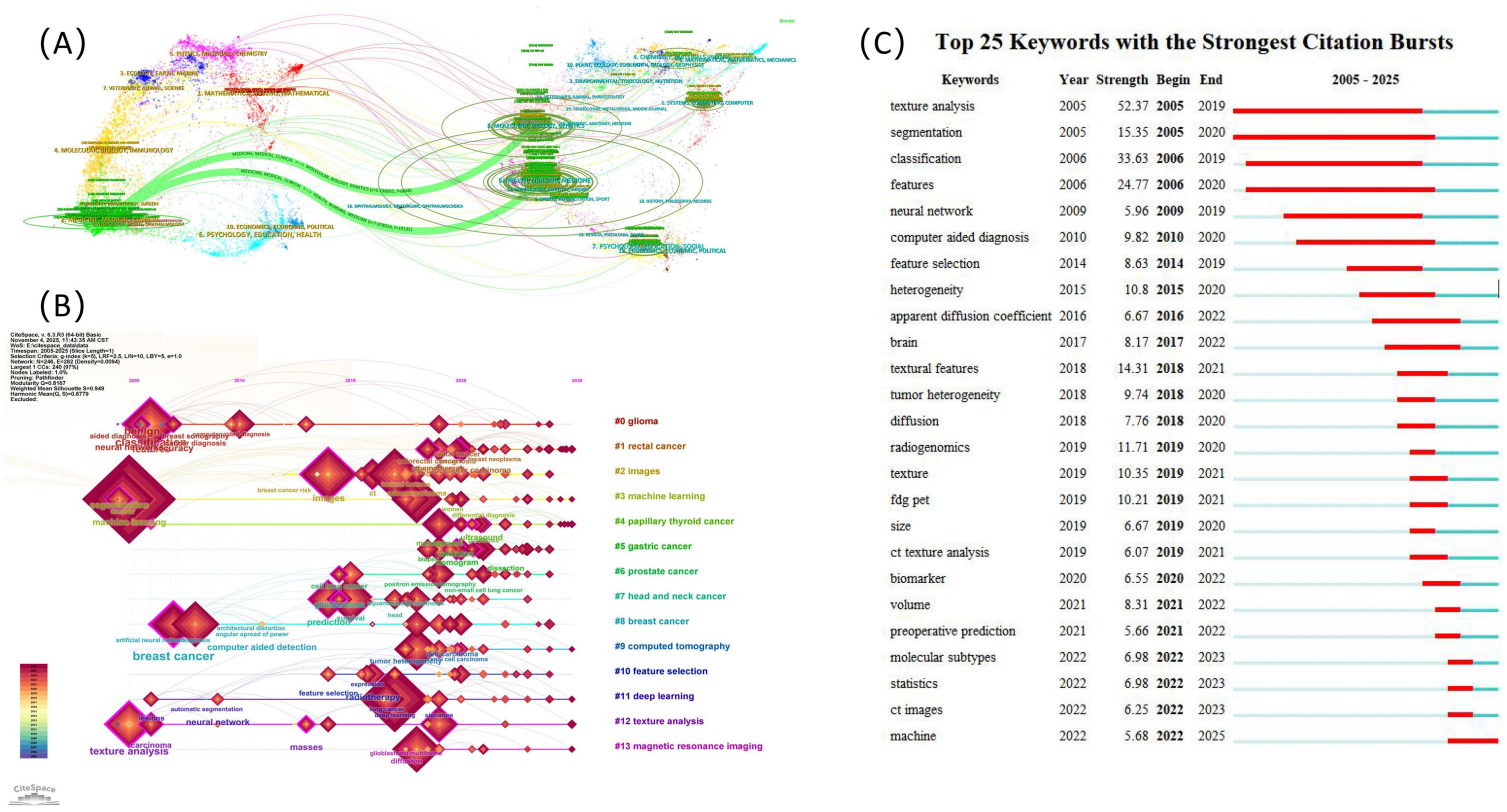
Disciplinary analysis of the research field.

A double-plot overlay shows the interjournal citation network, focusing on applying and integrating deep learning in tumour radiomics (Figure 3A). Each point corresponds to a journal, and the connection curves show citation relationships, emphasising interdisciplinary trends and citation development. The double-plot overlay method for journal analysis offers a compelling visualisation of the shift in research activity centres and the classification of journals across different academic disciplines. This double-plot overlay shows the interdisciplinary citation network for applying and integrating deep learning in tumour radiomics. The left side represents the science journal group, covering mathematics and molecular science, and the right side represents the basic science journal group, including molecular biology and physical chemistry, with the green line reflecting clinical translation trends.

CiteSpace subject clustering analysis revealed that Deep Learning formed 13 distinct research branches in the field of oncology (Figure 3B): #0Glioma, #1 Rectal Cancer, #2 Image Processing, #3 Machine Learning, #4 Papillary Thyroid Carcinoma, #5 Gastric Cancer, #6 Prostate Cancer, #7 Head and Neck Cancer, #8 Breast Cancer, #9 Computed Tomography, #10 Feature Selection, #11 Deep Learning, and #12 Texture Analysis. The network structure shows clear spatiotemporal evolution characteristics. The large node group in the lower-left corner, represented by journals in Engineering Medicine and Nuclear Medicine Radiology, constitutes the traditional research core and reflects the technological foundation of this field.

This study reveals the evolutionary trajectory of research hotspots in this field based on CiteSpace’s citation burst detection of literature from 2005 to 2025 (Figure 3C). The keyword with the highest intensity is “texture analysis” (52.37), with its burst period lasting until 2019. Early hotspots focused on basic methods such as “segmentation” and “classification,” while the research focus subsequently shifted to in-depth clinical analyses such as “heterogeneity” and “radiogenomics.” Recently, the burst of keywords such as “molecular subtypes” and “machine” indicates that the research frontier has entered the stage of precision medicine exploration based on machine learning. The overall trend reflects the deepening research process from methodological innovation to clinical translation and from morphological characterisation to molecular mechanisms.

### 3.3 Machine learning identifies 18 research topics in the field

**Figure 4:**
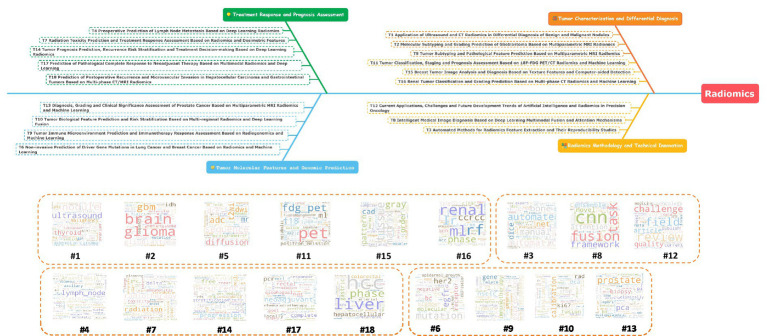
Topic modeling results of the research field.

The application and fusion of deep learning in tumour radiomics can be divided into four core research categories based on the LDA topic model analysis, with a total of 18 topics, reflecting the systematic and multidimensional features of this field (Figure 4).

LDA topic modelling analysis identified 18 core research topics associated with deep learning in tumour radiomics, which were summarised into four major application directions. The focus was on tumour feature description and differential diagnosis, encompassing six themes (T1, T2, T5, T11, T15, and T16). These themes included breast tumour image analysis and diagnosis based on texture features and computer-aided detection; molecular subtyping and grading prediction of gliomas based on multiparametric MRI radiomics; tumour subtyping and pathological feature prediction based on multiparametric MRI radiomics; application of ultrasound and CT radiomics in the differential diagnosis of benign and malignant nodules; classification and grading prediction of kidney tumours based on multiphase CT radiomics and machine learning; and tumour subtyping, staging, and prognostic assessment based on 18F-FDG PET CT radiomics and machine learning. The treatment response and prognostic assessment direction includes five themes (T4, T7, T14, T17, T18), which, respectively, predict postoperative recurrence and microvascular invasion of liver cancer and gastrointestinal tumours based on multiphase CT MRI radiomics; tumour prognostic prediction, recurrence risk stratification and treatment decision-making based on deep learning radiomics; prediction of pathological complete remission of neoadjuvant therapy based on multimodal radiomics and deep learning; prediction of radiotherapy toxicity and efficacy assessment based on radiomics and dosimetry characteristics; and preoperative prediction of lymph node metastasis based on deep learning radiomics. The tumour molecular characteristics and genomic prediction direction also included four themes (T6, T9, T10, and T13), focusing on the diagnosis, grading, and clinical significance assessment of prostate cancer based on multiparameter MRI radiomics and machine learning; non-invasive prediction of driver gene mutations in lung cancer and breast cancer based on radiomics and machine learning; prediction of the tumour immune microenvironment and evaluation of immunotherapy efficacy based on radiogenomics and machine learning; and prediction of tumour biological characteristics and risk stratification based on the fusion of multiregional radiomics and deep learning. Radiomics Methods and Technological Innovation direction also includes three themes (T3, T8, and T12): focusing on automated methods for radiomics feature extraction and their reproducibility research; intelligent medical image diagnosis based on deep learning multimodal fusion and attention mechanisms; and the current status, challenges, and future development trends of applying AI and radiomics in precision oncology.

The distribution of topics shows a clear research pattern in this field, forming a collaborative development trend with “tumour diagnosis” as the core, expanding in directions to “prognostic management” and “mechanism exploration,” and driven by “technological innovation” at the underlying level. This fully reflects the broad application prospects and huge potential of radiomics for precise oncological diagnosis and treatment.

### 3.4 Analysis of topic evolution and group development trends

**Figure 5:**
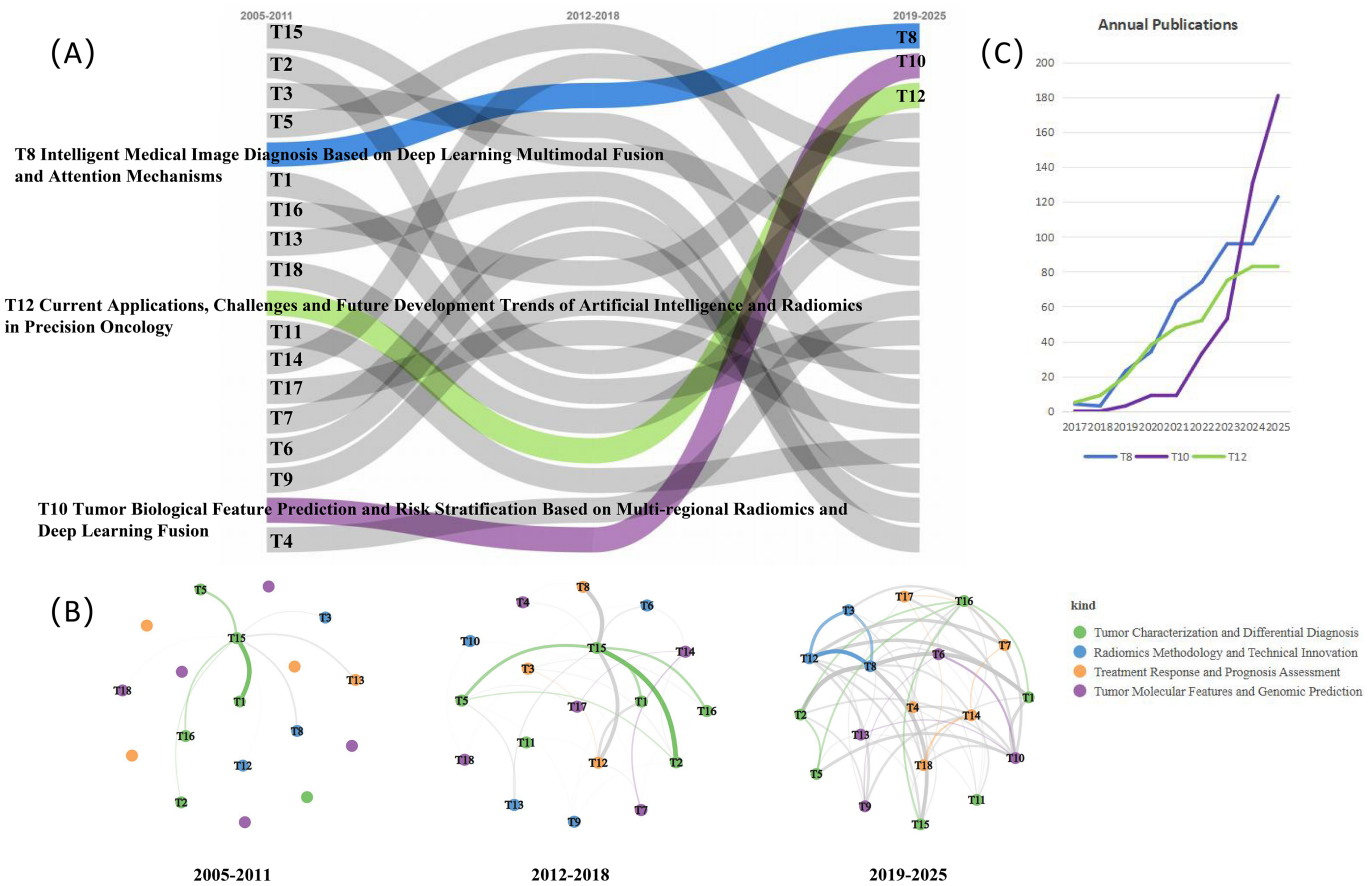
In-depth analysis of topic modeling results.

Based on the TSR analysis (Figure 5A), the research field shows a clear trend in thematic evolution, with the ranking changes of the three core themes reflecting the development trajectory of the field.

Theme evolution analysis based on TSR scores revealed that the research fields of deep learning and radiomics fusion underwent significant theme evolution between 2005 and 2025. Intelligent medical image diagnosis based on deep learning multimodal fusion and attention mechanisms (T8) went from 5th place in 2005–2011, to 3rd place in 2012–2018, and finally to 1st place in 2019–2025. Its network relationships evolved from a simple connection with breast tumour imaging analysis (T15) in the early stage (weight 6), establishing connections with multiple themes in the middle stage (weight 32), and becoming the core of the network from 2019 to 2025 (weighted degree 1,030), especially forming strong connections with T15 (weight 106), T12 (weight 116), and T2 (weight 126), indicating that multimodal fusion technology is widely used in various types of tumour research.

Tumour biological feature prediction based on the fusion of multiregional radiomics and deep learning (T10) has undergone a dramatic change from 17th (2005–2011) to 18th (2012–2018) and then to 2nd (2019–2025), reflecting the typical trajectory of explosive applications after technology matures. It formed a close relationship with risk stratification (T17, weight 70) and postoperative recurrence prediction (T18, weight 60) between 2019 and 2025, indicating that this method is reshaping the tumour prognostic assessment system.

The application of AI and radiomics in precision oncology (T12) rose from 10th place (2005–2011) to 3rd place (2019–2025). Its strong connection with methodological innovation (T8, weight: 116) and glioma subtyping (T2, weight: 66) shows the transformation of this field from conceptual exploration to practical implementation.

The evolutionary trajectory of these three topics reveals a paradigm shift in this field from single-modal analysis to multimodal fusion and from basic diagnosis to biomarker prediction and precision medicine.

Four major research clusters were formed based on a weighted analysis of the intertopic relationships from 2005 to 2025 (Figures 5B,C).

The tumour feature description and differential diagnosis cluster (T1, T2, T5, T11, T15, and T16) shows a developmental path from basic to refined. In the early stage (2005–2011), T15 (breast tumour imaging analysis) was the absolute centre (weight 52), absorbing contributions from other themes; in the middle stage (2012–2018), T15 and T2 (glioma subtyping) formed a dual-core structure (weight 18); and in the late stage (2019–2025), a more balanced network formed within the cluster. Notably, T2 and T5 (tumour subtyping and pathological feature prediction) established a strong connection in 2019–2025 (weight 50), whereas T2 also established an important connection with T12 (weight 66), indicating that glioma subtyping research is adopting more advanced AI technology. T16 (renal tumour classification) has recently emerged, and its strong relationship with T10 (weight, 76) reflects the cross-application of multiorgan research methods.

The radiomics methods and technology innovation clusters (T3, T8, and T12) have experienced significant growth and have also become bridges connecting other clusters. T8 (deep learning multimodal fusion) established a strong connection with T15 between 2012 and 2018 (weight 16); and between 2019 and 2025, it developed into a central connection point with all topics, forming a strong triangular relationship network with T2 (weight 126), T1 (weight 116), and T12 (weight 116), indicating that multimodal fusion methods have become the technical foundation for various types of tumour research. The relationship between T12 (AI applications and challenges) and T7 (radiotherapy toxicity prediction) first appeared between 2012 and 2018 (weight 4) and was significantly strengthened between 2019 and 2025 (weight 92), indicating that AI is actively used in optimising radiotherapy planning, and this technology transformation path is clearly visible.

The tumour molecular characterisation and genomic prediction cluster comprising T6, T9, T10, and T13 showed the most significant leaps. T10 jumped 15 places in its own ranking between 2019 and 2025 and formed strong multilateral connections with T5 (weight 80), T16 (weight 76), and T17 (weight 70). The relationship between T10 and T4 (lymph node metastasis prediction) (weight 68) reflects the value of deep learning in solving challenging clinical problems. The strong connection between T9 (tumour immune microenvironment prediction) and T14 (tumour prognosis prediction) (weight 66) reveals the trend of integration between the immune microenvironment and prognostic assessment research, which is of great significance in the era of immunotherapy.

The treatment response and prognostic assessment clusters (T4, T7, T14, T17, and T18) evolved from an early fragmented state into a tightly connected network. T14 began to show potential between 2012 and 2018, and by 2019–2025, it formed a strong connection with T18 (prediction of postoperative recurrence of liver cancer and gastrointestinal tumours) (weight 50). Simultaneously, its close relationship with T9 (weight 66) and T11 (PET CT radiomics and machine learning) (weight 46) indicates the importance of multimodal data integration in prognostic assessment. The connection established between T7 (prediction of radiotherapy toxicity) and T17 (assessment of neoadjuvant therapy) between 2019 and 2025 (weight 28) reflects the interoperability and sharing of treatment response assessment methodologies.

### 3.5 Temporal evolution trajectory analysis of important topics

**Figure 6:**
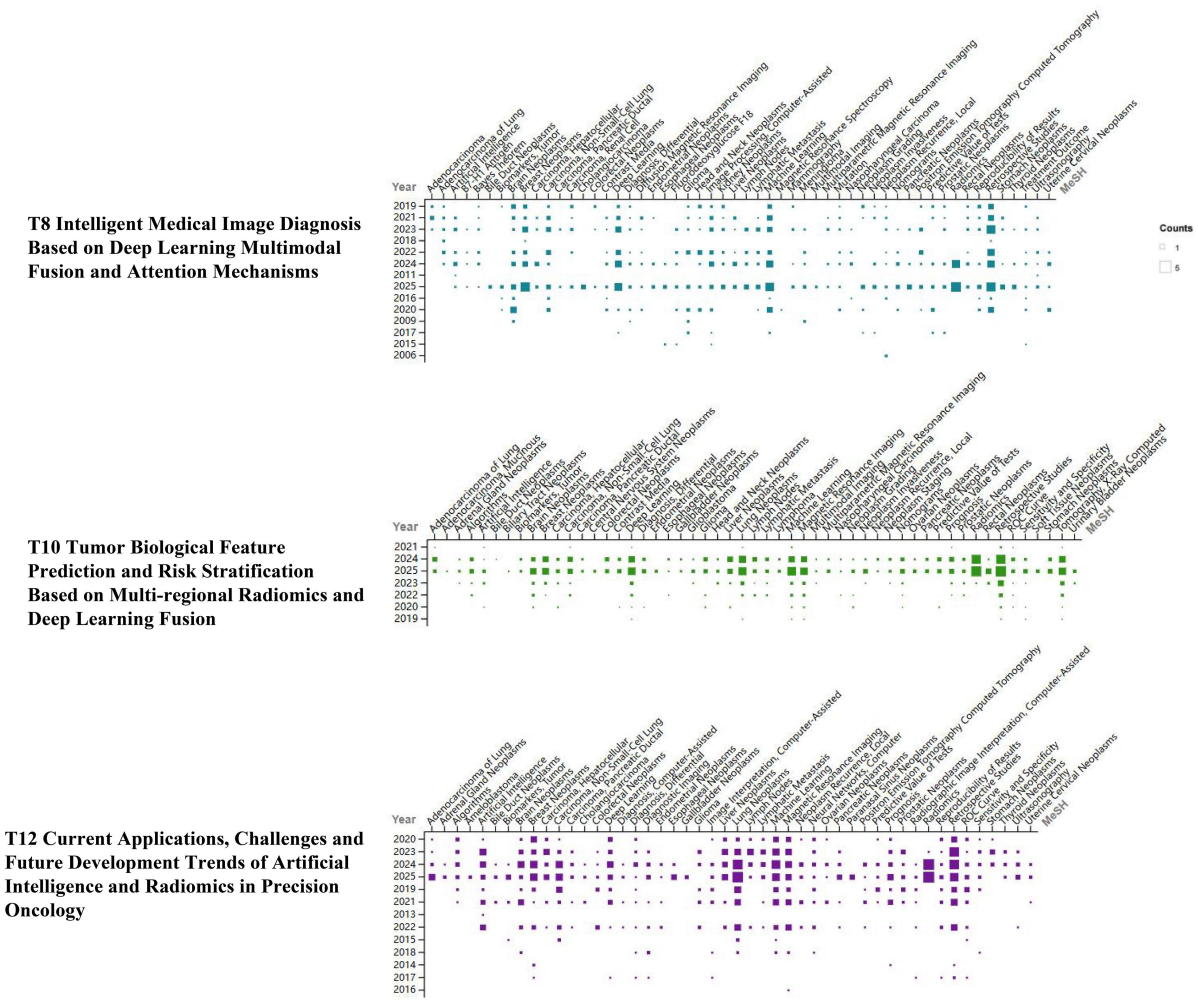
MeSH term analysis.

We analysed the MeSH terminology data from the relevant literature on high-TSR-ranked topics in this field (Figure 6) to reveal the evolution of each of these three topics:

The MeSH term evolution of T8-based deep learning multimodal fusion and attention mechanisms in intelligent medical image diagnosis reflected the rapid development trajectory of the field. In the early stage (2006–2015), research in this field was relatively scattered, mainly focusing on traditional imaging analyses of specific diseases, including brain neoplasms and meningiomas, with relatively basic techniques, such as applying Ultrasonography and Contrast Media. The mid-stage (2016–2020) showed a significant technological transformation, with an increase in machine-learning-related research (eight times in 2019 and 13 times in 2020), and the term Deep Learning began to appear (first in 2017, four times in 2019, and two times in 2020). The research scope has also expanded to include other types of tumours, including carcinoma, non-small cell lung cancer, breast neoplasms, and head and neck neoplasms. The increased frequency of image processing and computer-assisted image processing indicates a deepening application of algorithms in medical image processing. The recent period (2021–2025) has witnessed explosive growth in this field, with significant increases in technology and applications. The frequency of Deep Learning has risen sharply (from seven times in 2021 to 25 times in 2025), and research associated with AI continues to increase (three times in 2021 and seven times in 2024). The sudden emergence of radiomics as a field between 2024 and 2025 has been noted (26 and 38 times, respectively), indicating that quantitative image analysis methods are becoming a research hotspot. Multimodal Imaging emerged in 2025, reflecting recent research trends in multimodal fusion. Application areas have become more diversified with a significant increase in research on various tumours, including breast, pancreatic, and stomach neoplasms. Methodologically, the increased use of Bayes’ theorem and nomograms indicates an increasingly sophisticated predictive model. The number of retrospective studies continues to grow (from 12 in 2019 to 33 in 2025), showing researchers’ greater use of historical data to validate algorithm performance. Overall, this topic has evolved from traditional medical image analysis to deep-learning-based intelligent diagnostic systems with increasingly complex technologies and expanding clinical applications. Particularly, multimodal data fusion and radiomics methods are the latest developments in this field, providing more powerful decision-support tools for precision medicine.

The evolution of MeSH terms for T10-based tumour biological feature prediction and risk stratification using multiregional radiomics and deep learning reflects the early stage (2019–2020), when research in this field mainly focused on a few common tumour types (including brain, liver, and lung cancers) using basic machine learning and deep learning techniques for preliminary exploration. The number of studies was limited, and conventional CT and MRI were mainly used for imaging. The mid-stage (2021–2022) witnessed an initial expansion in the research scope, covering more tumour types, including non-small cell lung, prostate, and oesophageal cancers. During this stage, technical methods began to diversify, the frequency of deep learning applications increased (from single digits to five times), research designs gradually became more standardised, and the number of retrospective studies increased significantly. The recent stage (2023–2025) has witnessed explosive growth, especially in radiomics studies, which have rapidly increased from two in 2023 to 60 in 2024 and 72 in 2025. The applications of deep and machine learning have also grown exponentially, reaching 42 and 54 times, respectively, in 2025. Research subjects have expanded comprehensively to cover almost all major tumour systems, including lung tumours (including lung adenocarcinoma), brain tumours (such as glioma and glioblastoma), digestive system tumours (such as liver cancer, colorectal cancer, gastric cancer, and pancreatic tumours), and female reproductive system tumours (including breast and ovarian cancers). Furthermore, applying multimodal and multiparameter imaging techniques has significantly increased, reflecting the need for more refined clinical information. Research objectives have also shifted from early simple classification to more clinically valuable directions, including tumour invasiveness assessment (15 times in 2025), biomarker identification and validation, lymph node metastasis prediction, local recurrence assessment, and prognostic prediction. Notably, the application of clinically relevant indicators, such as nomograms, has increased significantly (reaching 16 times in 2025), with a corresponding increase in receiver operating characteristic (ROC) curves and sensitivity-specificity analyses, indicating that the research is moving towards clinical application. In summary, this topic has evolved from proof-of-concept to diversified applications and then to clinical translation-oriented approaches. Integrating radiomics and deep learning has become the mainstream technical route for predicting and stratifying tumour features. Research subjects and objectives have shown a trend towards diversification and refinement, reflecting the in-depth practice of precision medicine for tumour diagnosis and treatment.

The MeSH term evolution of the application status, challenges, and future development trends of T12 AI and radiomics in precision tumour diagnosis and treatment reflects that the application of AI in tumour diagnosis and treatment in the early stage (2013–2017) was in the exploratory stage. Research has mainly focused on specific tumour types, including breast and non-small cell lung cancers, with relatively limited technological applications and a small number of related studies. This period is the nascent stage of the field, which laid the foundation for its subsequent development. The mid-stage (2018–2021) showed a clear trend in technological expansion and diversified applications. Research subjects have expanded from single to multiple tumour types, such as brain tumours, hepatocellular carcinoma, and pancreatic cancer. Regarding technology, the application frequency of deep learning and machine learning has increased significantly, and multimodal imaging research, including MRI, has become a popular topic. Research methods have become more standardised during this period, and the number of retrospective studies has increased, indicating that the field is gradually maturing. The recent period (2022–2025) has witnessed the rapid development of radiomics, with its frequency surging from one occurrence in 2023 to 30 occurrences in 2025, becoming a core keyword in the field. The application of AI technology in tumour diagnosis and treatment has deepened comprehensively, with machine-learning-related research peaking in 2024 (19 occurrences). Research subjects have become more diversified, with lung tumour research being the most active (28 occurrences in 2025), whereas research on breast, liver, and oesophageal tumours continues to grow. The research focus has expanded from early diagnosis to prognostic prediction, recurrence prediction, and biomarker identification, reflecting a trend towards greater precision and personalisation in the field. Notably, the reproducibility of research methods and the reliability of results have received continuous attention throughout the research cycle, reflecting the field’s emphasis on scientific rigour. Overall, AI and radiomics have shown a developmental trajectory in the field of precision oncology, progressing from initial exploration to widespread application and then to deep integration. In the future, they are expected to be essential in early cancer diagnosis, precise subtyping, prognostic assessment, and treatment decisions.

### 3.6 Predicting future trends with emergence detection algorithms

**Figure 7:**
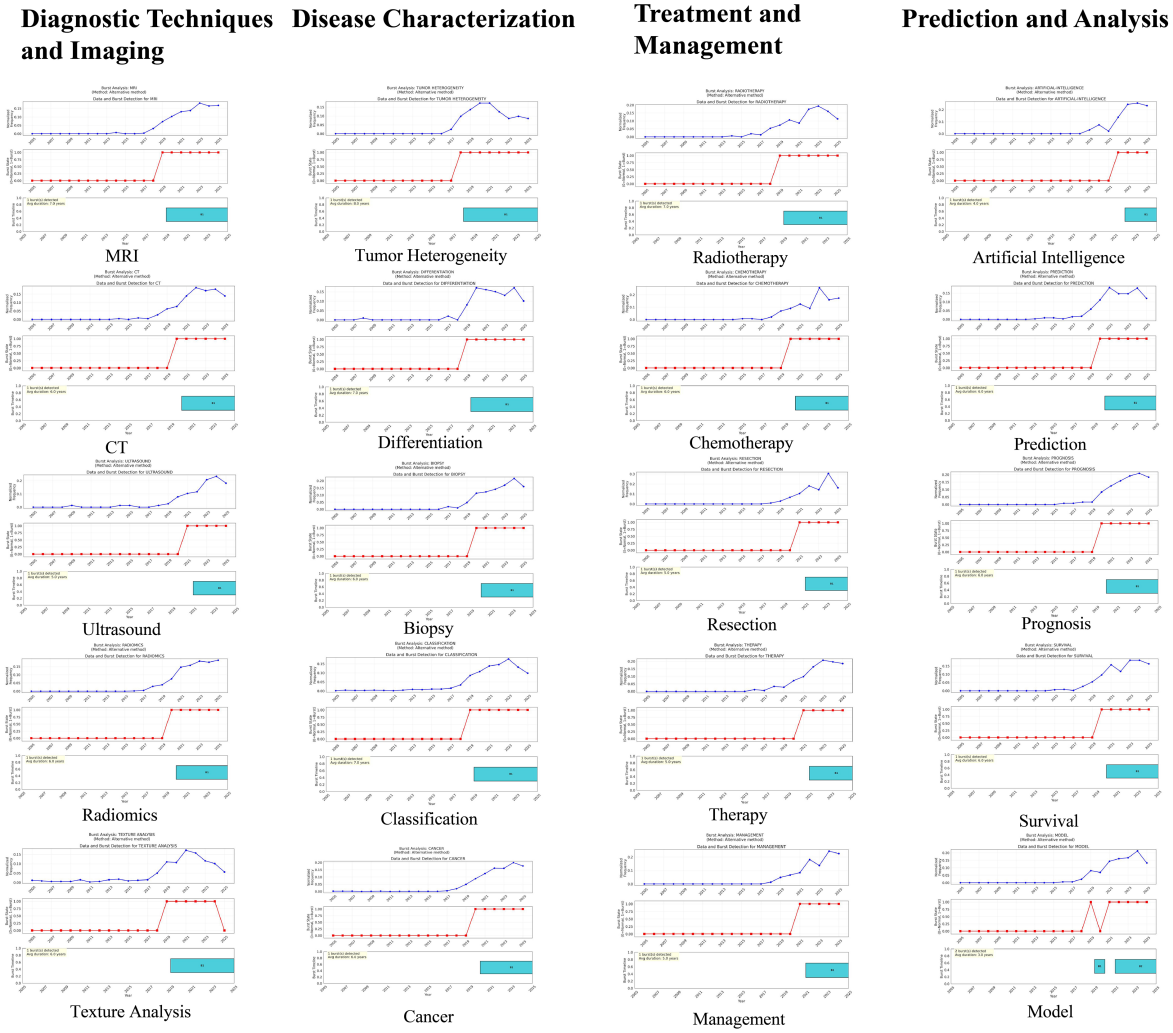
Future research trend prediction based on burst_detection algorithm for keywords in the field.

We conduct a time-series analysis of keywords acquired between 2005 and 2025. We aimed to predict research trends for these entities by applying the Burst Detection algorithm (Figure 7). The data preprocessing steps included normalisation and frequency thresholding to retain only entities with frequencies above the average. After these data-cleaning procedures, 20 named entities with significant research potential were identified. We used a dual-axis line chart to visually represent the results to predict the relevant keywords. The graph simultaneously presented the original and fitted publication trends, effectively capturing the evolutionary patterns and future development directions of these entities in academic research. Based on the semantic clustering and burst pattern analysis, four main research directions were identified: diagnostic technology and imaging, disease characterisation, treatment and management, and prediction and analysis.

Precision oncology diagnosis and treatment based on medical imaging are core directions in modern oncology research. Traditional imaging examinations, such as CT, MRI, and ultrasonography, are essential in tumour detection, localisation, and staging. However, these routine examinations rely mainly on qualitative assessments of morphological characteristics, making it difficult to comprehensively reflect the complex spatial information within the tumour. Tumours show high heterogeneity at the microscopic level, which is closely associated with the degree of cell differentiation, treatment resistance, and poor prognosis. Traditional biopsy is the gold standard for pathology; nevertheless, its invasiveness and sampling errors limit its ability to comprehensively assess the overall heterogeneity of tumours. Radiomics and texture analysis technologies have provided new avenues for addressing this challenge. These technologies extract various quantitative features from high-throughput medical images and transform the image data into a mineable high-dimensional feature space. These features, especially texture features, can non-invasively quantify heterogeneity within tumours, thereby enabling a more objective classification of tumour malignancy, genotyping, and differentiation grade. This provides strong data for precise cancer diagnosis, efficacy prediction, and survival prognostic assessment. Currently, radiomics research is deeply integrated with AI, particularly deep learning. Deep learning models can automatically learn discriminative feature representations from raw images, thereby avoiding the limitations of traditional manual feature extraction. Researchers can non-invasively predict tumour sensitivity to radiotherapy or chemotherapy before treatment and select the optimal personalised therapy for patients by constructing AI-based predictive models, thus achieving more precise clinical management. Furthermore, AI models can accurately predict the extent of tumour invasion and histological grade preoperatively, providing crucial decision-making support for surgical resection plans and ultimately driving the tumour diagnosis and treatment model towards precision and personalisation. In summary, integrating deep learning and radiomics has profoundly changed the paradigm of tumour imaging data analysis and application, showing significant potential for improving the overall management of tumours.

## 4 Discussion

### 4.1 Principal findings

In this study, we found that over the past two decades, and especially since 2020, deep learning-enabled tumour radiomics has experienced explosive growth: annual publications rose from 253 in 2019 to 1,021 in 2024, signalling an unprecedented phase of activity. This surge is owing to the simultaneous maturation of computational hardware, deep-learning algorithms, and the availability of large-scale multi-parametric imaging repositories (Litjens et al., 2017; Esteva et al., 2021). Country-level contribution analysis revealed a bipolar structure dominated by China and the United States. China now supplies over two-thirds of the global output (692 papers in 2025), yet the USA remains the principal hub of international collaboration, registering the highest total link strength (Chen et al., 2023). China’s comparatively low outward collaboration index indicates room for deeper global integration. Institutional co-authorship maps are dominated by Chinese universities and academies, while overseas centres participate only peripherally, underscoring the prevailing pattern of intra-national networks. A journal cocitation map centred on Cancers, European Radiology, Institute of Electrical and Electronics Engineers transactions on medical imaging, and Medical Image Analysis bridges radiology, computer science, and oncology and constitutes the interdisciplinary backbone of knowledge diffusion (Kumar et al., 2012; Gillies et al., 2016). As shown in Figure 2B, China dominates in raw publication output yet exhibits relatively low international collaboration link strength, whereas the United States and several European countries serve as central hubs of collaboration. This pattern likely reflects structural and linguistic factors (Adams and Gurney, 2018; Marginson, 2018). China’s vast domestic research infrastructure and large, centralized patient cohorts facilitate high-output intramural research (Ouyang et al., 2025). In contrast, the US and European nations operate within long-established multilateral funding frameworks and shared regulatory standards that inherently promote international co-authorship (Almeida et al., 2022). Encouraging multinational consortia with explicit data-sharing protocols could help bridge this gap and enrich global knowledge exchange.

An LDA topic model resolved 18 core topics into four macro-domains: (i) tumour characterisation and differential diagnosis (six topics), (ii) therapy response and prognostic evaluation (five topics), (iii) molecular profiling and genomic prediction (four topics), and (iv) methodological and technical innovation (three topics) (Blei et al., 2003; van Griethuysen et al., 2017). This taxonomy shows a systematic, multi-layered research arena that extends from diagnostic imaging toward prognostic management and mechanistic exploration, while using technological innovation as its engine (Lambin et al., 2017). Diagnostic themes remain the most frequent; however, topics associated with outcome prediction and molecular surrogates have expanded fastest, mirroring the field’s evolution from morphological triage to precision-oncology decision support (Aerts et al., 2014; Bi et al., 2019).

Burst-detection and temporal-topic modelling show that early hotspots such as “texture analysis” and “segmentation” have given way to “intra-tumour heterogeneity” and “radiogenomics,” and, more recently, to “molecular subtyping” and “machine-learning-driven precision medicine” (Hosny et al., 2018; Park et al., 2020). This trajectory reflects a deepening transition from methodological invention to clinical translation and from phenotypic to genotypic characterisation (Pinker et al., 2018). Co-authorship and topical-cluster analyses indicate that while the USA–China axis dominates scientific production, thematic convergence is tight and multimodal fusion technology (Topic 8) functions as a cross-cluster bridge (Liu et al., 2019). Plausible drivers include attention-based deep architectures that enhance feature expressivity, integrative multi-parametric pipelines that improve model robustness, and growing clinical demand for noninvasive biomarkers (Vaswani et al., 2017; Zhou et al., 2021). Future agendas should prioritise stronger transnational consortia, the standardisation of algorithms, and prospective clinical validation (Mongan et al., 2020). However, bibliometric trends alone do not guarantee clinical readiness. Major barriers—including limited external validation, data heterogeneity, reproducibility concerns, and regulatory constraints—must be systematically addressed before these technologies can be considered for bedside implementation (Sivakumar et al., 2025; Xu et al., 2025; Borra et al., 2026).

### 4.2 Temporal dissection of topic evolution

TSR analysis delineates three successive phases that mirror paradigm shifts in the discipline.

#### 4.2.1 Early phase (2005–2011)

Research centred on methodological groundwork. Breast tumour image analysis (T15) occupied the dominant topological position (weighted degree = 52) with few inter-topic connections (Depeursinge et al., 2014). Texture extraction and conventional machine learning classifiers were the mainstays, and dual-map overlays showed citation flows confined to physics and engineering journals (O'Connor et al., 2017), indicating limited interdisciplinary dialogue.

#### 4.2.2 Middle phase (2012–2018)

The advent of deep learning reconfigured the thematic landscape. Multimodal fusion (T8) increased from rank 5 to rank 3, forging strong links with T15 (weight = 32). Concurrently, glioma subtyping (T2) has emerged, forming a dual-core structure with T15 and widening its oncological applications (Zhou et al., 2018). Citation pathways have begun to reach clinical journals, foreshadowing translational momentum (Parmar et al., 2015).

#### 4.2.3 Recent phase (2019–2025)

An inflextion point will occur. Multimodal fusion (T8) became the top-ranked topic, while tumour biological-feature prediction (T10) rose from 18th to 2nd place, and AI applications (T12) entered the top three. A densely connected network developed, with T8–T2 (weight = 126) and T8–T12 (weight = 116) dyads, exemplifies intracluster cohesion (Lao et al., 2017). Research pivoting from single-modality diagnosis to multi-parametric, prognostic, and mechanistic modelling underscores the penetration of precision oncology principles (Bodalal et al., 2019). Collectively, evolution depicts a transition from technology-driven to clinic-driven investigation, with multidisciplinary convergence as the new norm.

### 4.3 Deep-dive analysis of pivotal topics

We dissected the three highest-impact topics identified by TSR metrics to illuminate technical maturation and clinical embedding.

T8—“Deep-learning-based multimodal fusion with attention mechanisms for intelligent tumour imaging diagnosis.” MeSH-term evolution shows early dispersal (2006–2015) of brain tumours and ultrasonography, followed by mid-term concentration (2016–2020) of machine and deep-learning applications to lung and breast cancers (Shen et al., 2017). The latest interval (2021–2025) shows explosive growth: “deep learning” occurrences rose from seven (2021) to 25 (2025), while “radiomics” and “multimodal imaging” became dominant nodes. This signals the migration from proof-of-concept to deployment, with attention-based architectures enhancing interpretability and generalisability across tumour types.

T10—“Multi-regional radiomics fused with deep learning for biological-feature prediction and risk stratification.” Initial efforts (2019–2020) targeted hepatocellular and lung carcinomas. Methodological diversification will appear during 2021–2022, with deep learning gaining traction. The latest period (2023–2025) showed a 72-count burst for “radiomics” and expanded anatomical coverage (Trebeschi et al., 2021). End-points have moved beyond classification toward invasion assessment and nodal metastasis prediction, accompanied by the increased use of nomograms and ROC/Area Under the Curve reporting markers of translational utility (Iasonos et al., 2008). This topic embodies the potential of the radiomics–deep learning synergy to decode intratumor heterogeneity and biological aggressiveness (Grossmann et al., 2017).

T12—“AI and radiomics in precision oncology: current status, challenges and future directions.” Early discourse (2013–2017) focused on single-tumour feasibility; the intermediate phase (2018–2021) witnessed technique proliferation and multi-parametric imaging. The latest epoch (2022–2025) is characterised by “radiomics” centrality (30 occurrences in 2025) and an expanded remit covering prognostic biomarkers and reproducibility crises (Fogel and Kvedar, 2018). Persistent emphasis on “reproducibility” indicates community awareness of robustness gaps (Mongan et al., 2020). This theme captures AI’s graduation from ancillary tools to decision-support systems, while foregrounding outstanding issues of standardisation, ethics, and clinical integration (Bi et al., 2019; Topol, 2019).

### 4.4 Emerging methodological frontiers in deep learning-driven radiomics

The topic modelling results presented in this study identify “methodological innovation” as one of the four pillar domains of the field. A number of emerging methodological directions are reshaping the frontier and deserve explicit consideration for future radiomics research.

First, transformer-based architectures, originally developed for natural language processing, have been rapidly adapted to medical image analysis. Vision Transformers and hybrid CNN-transformer models exploit self-attention mechanisms to capture long-range spatial dependencies that are often missed by purely convolutional approaches (Shamshad et al., 2023; Zhou et al., 2023; Kim et al., 2025). In tumour radiomics, these architectures have shown particular promise in tasks requiring global context, such as whole-tumour characterisation and multi-regional feature integration, and they align closely with the multimodal fusion trends captured by Topic 8.

Second, the rise of foundation models and transfer learning is beginning to address one of the central bottlenecks in radiomics: the scarcity of large, well-annotated clinical datasets. Foundation models pre-trained on extensive and diverse imaging repositories can be fine-tuned for downstream radiomic tasks with comparatively limited data, offering a pathway toward improved generalisability across institutions, scanners, and acquisition protocols (Moor et al., 2023; Napravnik et al., 2025). This is especially pertinent given the strong but largely intra-national collaboration patterns observed in our institutional network analysis.

Third, although deep learning models are increasingly used for clinical endpoint prediction, the majority of published studies still report point predictions without accompanying confidence estimates. Uncertainty quantification methods—such as Monte Carlo dropout, deep ensembles, and Bayesian neural networks—provide voxel-level or case-level uncertainty maps that can flag ambiguous predictions for clinician review (Faghani et al., 2023; Huang et al., 2024; Lambert et al., 2024). The integration of such methods into radiomic workflows has the potential to enhance safety and trust.

Fourth, closely related is the growing adoption of Explainable Artificial Intelligence (XAI) techniques. Methods such as gradient-weighted class activation mapping, Shapley Additive Explanations, and attention map visualisation are being employed to verify that model decisions are grounded in biologically and clinically meaningful image regions (Nazir et al., 2023; Bhati et al., 2024; Fayyad et al., 2024). In the context of radiomics, XAI can help bridge the gap between deep-learned features and established radiomic feature categories, thereby strengthening both interpretability and clinical acceptance.

Collectively, these advances speak to the broader challenge of robustness and generalizability that will determine whether deep learning-based radiomics can transition from retrospective proof-of-concept studies to prospective clinical deployment (Begoli et al., 2019; Seoni et al., 2023; Xu et al., 2025). The strong cross-topic connectivity observed for T8 (multimodal fusion) and T12 (AI applications and challenges) in the recent period (2019–2025) suggests that the field is already moving in this direction. Making these methodological innovations a more explicit focus of future research—and of future bibliometric tracking—will be essential for realising the precision oncology promises outlined in this analysis.

### 4.5 Limitations

Our exclusive reliance on the Web of Science Core Collection may introduce a database-specific bias, particularly underrepresenting computer science and engineering-centric publications that are more commonly indexed in Scopus or IEEE Xplore. Given that the “Methodological Innovation” domain (encompassing Topics T3, T8, and T12) involves substantial algorithmic development, the omission of leading computational conferences and technical journals could influence the perceived weight and trajectory of this domain. Future bibliometric investigations would benefit from cross-database validation to capture the full spectrum of technical contributions driving this interdisciplinary field.

The analyses were terminated in 2025. Emergent themes, including generative-AI-driven radiomics, may not have accrued sufficient citation mass to rank highly. Therefore, prolonged observation windows are required.

LDA also has some limitations. Topic number specification, bag-of-words simplification, and manual labelling introduce subjective bias (Blei et al., 2003; Griffiths and Steyvers, 2004). Future work could integrate contextualised embeddings to improve semantic fidelity. Our LDA topic model was constructed solely from titles and abstracts, which may lack sufficient semantic depth to fully characterize complex methodological topics, potentially leading to under-specified topic granularity.

## 5 Conclusion

In summary, multi-dimensional analyses have suggested an emerging paradigm shift in deep learning-based tumour radiomics. Subsequent investigations should prioritise transcontinental consortia, multi-institutional data sharing, and rigorous prospective validation to support efforts toward the translation into precision oncology workflows.

## Data Availability

The original contributions presented in the study are included in the article/Supplementary material, further inquiries can be directed to the corresponding author.

## Footnotes

1
https://bibliometric.com/
