# Supplementary material for: Application and integration of deep learning in tumour radiomics: bibliometrics and visualisation analysis
Source: Front Artif Intell. 2026 Jul 29;9:1779682. doi: 10.3389/frai.2026.1779682 (PMC13461793; doi:10.3389/frai.2026.1779682)
Supplement: Supplementary file 1 [file Presentation_1.zip › Sep_Supplementary_Material/Supplementary Figure S1.pdf]

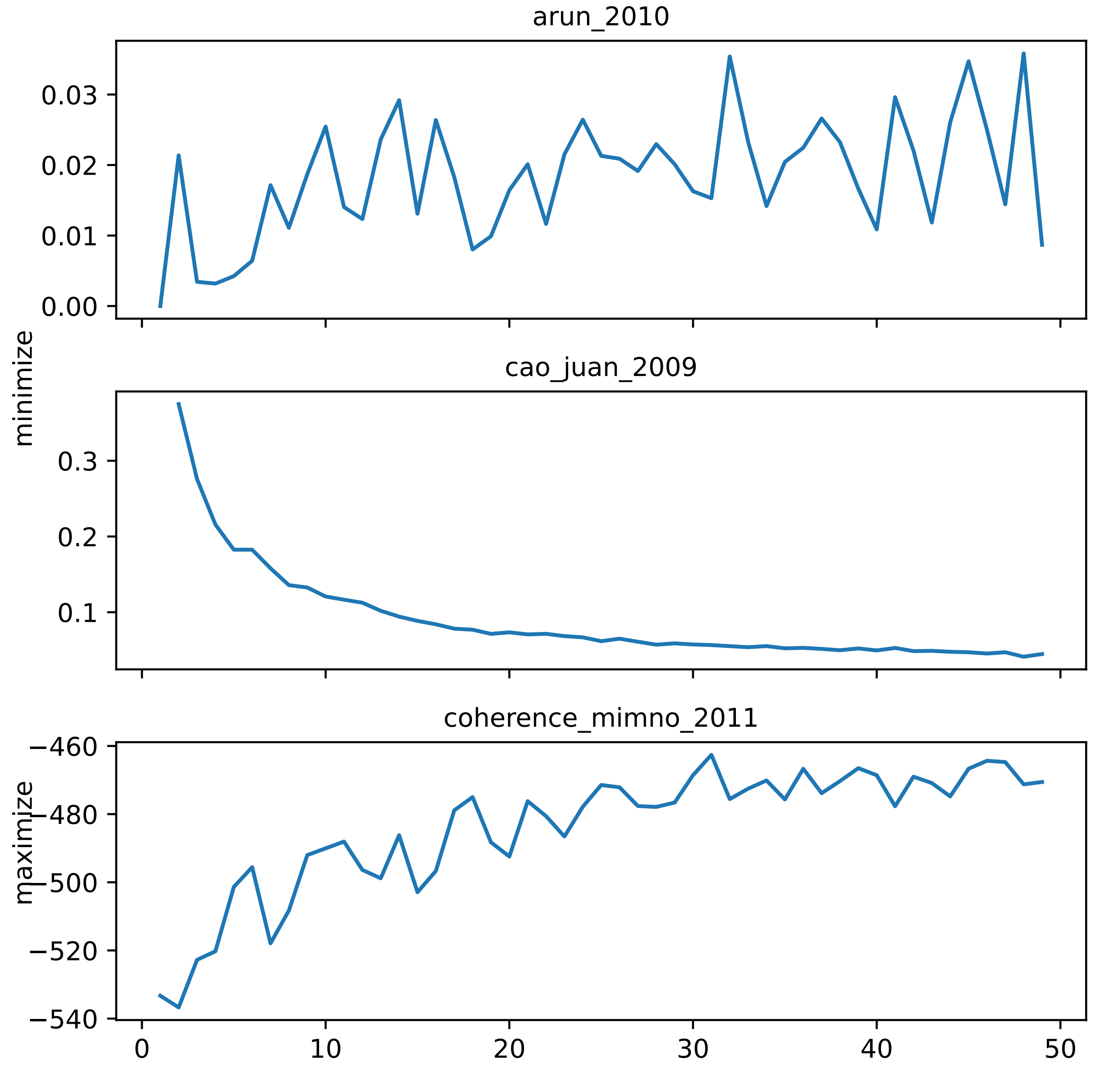

arun_2010
0.03
0.02
0.01
0.00
minimize
cao_juan_2009
0.3
0.2
0.1
coherence_mimno_2011
−460
−480
−500
−520
−540
maximize
0
10
20
30
40
50
