# Supplementary material for: Application and integration of deep learning in tumour radiomics: bibliometrics and visualisation analysis
Source: Front Artif Intell. 2026 Jul 29;9:1779682. doi: 10.3389/frai.2026.1779682 (PMC13461793; doi:10.3389/frai.2026.1779682)
Supplement: Supplementary file 1 [file Presentation_1.zip › Sep_Supplementary_Material/Supplementary_File1.docx]

Web of Science Search Strategy

**Database:** Web of Science Core Collection
**Search Date:** October 28, 2025

**Filters Applied:**

Document Type: Article

Time Span: All years (as of the search date)

**Search Fields:** Topic. Searches title, abstract, keyword plus, and author keywords.

**Query String:**

( ( (TS=("cancer" OR "neoplasm*" OR "tumor*" OR "tumour*" OR "malignanc*" OR "oncology")) AND TS=("deep learning" OR "convolutional neural network*" OR "CNNs" OR "artificial intelligence" OR "machine learning" OR "neural network*")) AND TS=("radiomic*" OR "radiogenomic*" OR "texture analysis" OR "imaging signature*" OR "imaging biomarker*" OR "image feature extraction"))
