# Supplementary material for: Application and integration of deep learning in tumour radiomics: bibliometrics and visualisation analysis
Source: Front Artif Intell. 2026 Jul 29;9:1779682. doi: 10.3389/frai.2026.1779682 (PMC13461793; doi:10.3389/frai.2026.1779682)
Supplement: Supplementary file 1 [file Presentation_1.zip › Sep_Supplementary_Material/Supplementary_File2.docx]

**Supplementary Text 1**: Topic Modeling Background

**Latent Dirichlet Allocation (LDA)**

Latent Dirichlet Allocation (LDA), a natural language processing technique, was used to discover latent topics. LDA divides the content of the text into three levels, namely document, topic, and words, and represents the distribution of topics in each document as:

$P(w|d)=P(w|t)\times P(t|d)$	(1)

where $w, t,$ and $d$ represent words, topics, and documents, respectively. This process can be further represented as:

$P(w_{m},z_{m},\theta_{m},\Phi|\alpha,\beta)=\Pi_{n=1}^{N_{m}}p(\Phi|\beta)p(\theta_{m}|\alpha)p(z_{m,n}|\theta_{m})p(w_{m,n}|\Phi,z_{m,n})$	(2)

where $N_{m}$ represents the length of document $m$, and $z_{m,n}$represents the topic generated by document $m$.

To obtain the final topic model result, we chose the Gibbs sampling method based on the Markov Chain Monte Carlo (MCMC) algorithm to estimate the parameters of the LDA model.Various criteria were used to determine the number of topics **(Supplementary Figure S1)**.

Raw data, article titles and abstracts, were imported into Microsoft Excel 2021 for preliminary organization. Frequency thresholds were established, common stop words removed, and specific stop words defined. LDA topic modeling and word cloud maps were generated using Python packages. Each topic was manually named based on the 10 most prominent articles and 20 topic terms.

**Number of Topics**

Although LDA probabilistic models demonstrate flexibility in describing topic correlations and can capture subtle differences and complex associations between topics, they all face the same challenge of determining the number of topics (parameter K). The selection of this parameter is crucial, as it directly affects the quality, granularity, and effectiveness of the topic structure ultimately extracted by the model. An inappropriately chosen K value may lead to topics that are either too broad and lack discriminability, or too detailed and difficult to generalize, thereby affecting the model's understanding and application to texts. This study employed the following methods to address the dilemma of topic number selection:

Pairwise Cosine Distance Method[1]

The pairwise cosine distance method, proposed by Cao et al. in 2009, is based on the core concept of utilizing the stability of topic models across different runs to evaluate the quality of the topic number (K). This method assumes that for a given dataset, if the selected number of topics is appropriate, then even when running the topic model multiple times, the resulting topic structures should be relatively stable, meaning that the topic distributions from different runs should be quite similar.

Specifically, the pairwise cosine distance method runs the topic model multiple times on the same dataset using the same topic number K. For the topic distribution obtained from each run, the method treats it as a vector and calculates the cosine distance between corresponding topics from any two runs. Since each run may yield topics in different orders, topic matching is required, typically by finding pairs of topics with the highest cosine similarity between two runs. After calculating cosine distances between all possible paired topics, these distances are averaged to obtain an indicator measuring model stability under that particular K value.

By testing different K values and calculating their corresponding average pairwise cosine distances, we can observe that as K gradually increases, model stability typically first improves, reaches a peak, and then begins to decline. The key assumption of this method is that when the average pairwise cosine distance reaches its maximum value, the corresponding K value is likely the optimal number of topics for the dataset. This is because at this point, the model can stably discover similar topic structures, avoiding both overly broad topics due to too small K values and overly fragmented topics due to too large K values.

Kullback-Leibler Divergence Method^[2]^

Arun et al. proposed a metric method based on symmetric Kullback-Leibler divergence. This method extracts certain "salient distributions" from the "document-topic matrix (M1)" and "topic-word matrix (Q)" - the two matrix factors obtained from LDA algorithm decomposition - and then computes the symmetric KL divergence between these distributions. The research observed that when the selected number of topics is not optimal, the computed KL divergence values are relatively high. However, when the number of topics approaches or equals the natural number of topics existing in the corpus, the KL divergence values exhibit a distinct "dip." This "dip" point is considered an indicator of the optimal number of topics.

The workflow of this method involves: running LDA models with different topic numbers K, obtaining corresponding matrix decomposition results, and calculating the corresponding symmetric KL divergence values. By observing the trend of KL divergence values as K changes, particularly by searching for that "dip" point, one can effectively identify the optimal number of topics for the corpus.

Model Consistency^[3]^

The "harmonic mean method" proposed by Mimno et al. centers on the core idea of determining the optimal K value by calculating data probabilities under models with different topic numbers and seeking the model with the highest probability value. Specifically, for a series of different K values, researchers train LDA models separately. For each trained model, the harmonic mean method attempts to estimate the probability of generating the currently observed data under that model. Since directly calculating the marginal likelihood of LDA models is typically difficult, this method adopts an approximation strategy using harmonic means to estimate this probability.

The harmonic mean calculation proceeds as follows: for each document, first calculate the log-likelihood value of generating that document under the model. Then, take the exponential of all documents' log-likelihood values to obtain the likelihood value for each document. Next, calculate the average of the reciprocals of these likelihood values, and finally take the reciprocal to obtain the harmonic mean likelihood estimate for the data under that model. This method assumes that when the model's topic number best matches the true latent structure of the data, the probability of that model generating the current data will also be highest. Therefore, by comparing harmonic mean likelihood estimates corresponding to different K values, one can select the K value that yields the highest harmonic mean likelihood estimate as the optimal number of topics.

This represents topic consistency, while being related to document likelihood values. By calculating harmonic mean likelihood estimates corresponding to different topic numbers K, we can evaluate the model's topic consistency and select the optimal K value, thereby ensuring that the model's topic number matches the true structure of the data.

**Supplementary Text 2**: Topic Significance Ranking (TSR)

To assess topic quality, the Topic Significance Ranking (TSR) score was used, following criteria proposed by AlSumait[4]. TSR is a novel unsupervised analysis of Probabilistic Topic Models (PTM). We applied a 4-phase Weighted Combination decision strategy to evaluate the inferred topics of PTM to evaluate their semantic significance. Topic significance is obtained by combining the information from the "multi-criteria measures" to form a single index of evaluation based on a "Weighted Linear Combination" (WLC) decision strategy. WLC is a widely used technique in the area of multi-criteria decision analysis. WLC assesses each topic by the following formula:

$A_{k}=\sum_{m=1}^{N_{m}} \Psi_{m}S_{m,k}$	(1)

where $N_{m}$ is number of different measures, $\Psi_{m}$ refers the weight of the measure in the total score, and $S_{m,k}$ is the score of $k$ th topic for the $m$ th measure.
A 4-phase weighted combination approach was implemented. $S_{m,k}$ and $\Psi_{m}$ scores were to be computed on different scales and criteria; therefore, measures are standardized before combination.
Phase 1: Standardization procedure

In the first phase, "standardization procedures" are performed to transfer each distance measure from its true value into two standardized scores (relative score of the distances and weight value).

To compute both the scores $S_{m,k}$ and weights $\Psi_{m}$, two standardized procedures are used for each of the criteria. The first, score is re-scaled based on the weight of each score against the total score overall topics and is expressed in Eq. (2). The second, score range procedure is followed based on the minimum and maximum values as scaling points for standardization and is given by Eq. (3).

$\begin{matrix} {\acute{C}1}_{k}^{m}=C_{k}^{m}\times\frac{\sum_{j=1, j\neq k}^{k} C_{j}^{m}}{\sum_{j=1}^{k} C_{j}^{m}} \\ \end{matrix}$	(2)

${\acute{C}2}_{k}^{m}=\frac{C_{k}^{m}-C_{\min}^{m}}{C_{\max}^{m}-C_{\min}^{m}}$	(3)

where $C_{\min}^{m}$ and $C_{\max}^{m}$ are the minimum and maximum distance values measured by the distance measure $m$ under the criterion $C$, and $m\in\{KL,COR,COS\}$, KL-Kullback-Leibler (KL) Divergence, COR-Correlation Coefficient, and COS-Cosine Dissimilarity are three distance measures.

Phase 2: Intra-criterion weighted linear combination.

To compute the rank significance, different measures are combined into a single figure. In this phase, topic ranking performs a WLC of the standardized score of the distance measures. The WLC score of criteria $C$ for topic $k$ is given by $\mathrm{Eq}.(4)$

$S_{k}^{C}=\frac{\acute{C}_{k}^{\mathrm{KL}}+\acute{C}_{k}^{\mathrm{COR}}+\acute{C}_{k}^{\mathrm{COS}}}{3}$	(4)

where $C_{k}^{\mathrm{KL}},C_{k}^{\mathrm{COR}}$, and $C_{k}^{\mathrm{COS}}$ are the standardized scores of the three distance measures.

Phase 3: Inter-criterion Weighted Combination

In phase 3, a Weighted Combination (WC) is done over the scores computed in phase two. Two different WC approaches are used to combine the scores and weights. The first approach is based on Eq. (2) and applies the standardized score of the background criterion as a weight for the Uniformity (u) and Vacuousness ($\nu$).

$\hat{S}_{k}=\hat{S1}_{k}^{b}\left( \acute{\Psi}_{u}{S1}_{k}^{u},+\acute{\Psi}_{v}{S1}_{k}^{v} \right)$	(5)

where $\Psi_{u}$ and $\Psi_{v}$ are the weights of the Uniformity and Vacuousness criteria in the score, respectively; $\xi_{1}^{b}$ is the weight of the topic background.
The second approach is performed based on the score range standardization procedure (Eq. 3). This is done by using the WLC in Eq. (1) as below:

$\hat{\Psi}_{k}=\Psi_{u}{S2}_{k}^{u}+\Psi_{v}{S2}_{k}^{v}+\Psi_{b}{S2}_{k}^{b}$	(6)

Phase 4: Topic Significance Score

The final rank is computed using the total topic score from Eq. (5) and the normalized weight from Eq. (6). Thus, the final rank of the topic is computed by:

$\mathrm{TS}R_{k}=\hat{\Psi}_{k}\times\hat{S}_{k}$	(7)

A significance ranking algorithm was applied on the topics discovered by LDA with $K$ set to 20. This technique has the potential to properly rank the topic based on its true significance. The rank of a topic depends on its distribution of background words. A topic with higher TSR describes the underlining themes better and presents the meaningful class semantics. The topics are ordered by their rank.

**Supplementary Text 3**: Burst Detection Algorithm

The Burst Detection algorithm proposed by Kleinberg[5]is a method for identifying periods of abnormally frequent event occurrences in time series data. It is based on the following assumption: time series data can be viewed as transitioning between multiple states, with each state corresponding to different target event probabilities. In this implementation, we consider two states: the baseline state and the burst state.

The baseline probability is defined as:

$p0=R/D$	(1)

where R is the total number of target events (the expression level of the specific gene being analyzed at each time point), and D is the total number of all events (the total expression level of all genes at each time point).

The burst state probability is defined as:

$p1=s*p0$	(2)

Where $s$is a customizable parameter that controls the difference between the burst state and the baseline state.

The algorithm determines the optimal state sequence by minimizing a cost function. The cost function consists of two parts:

1. Fitting cost ($\sigma$)：measures the difference between the observed proportion and the expected probability.

$\sigma(i,r_{t},d_{t})=-\ln[\left( \frac{d_{t}}{r_{t}} \right)p_{i}^{r_{t}}\left( 1-p_{i} \right)^{d_{t-r_{t}}}]$	(3)

2. Transition cost (τ)：measures the difficulty of transitioning from the previous state to the next state.

$\tau=\left( i_{\mathrm{next}}-i_{\mathrm{prev}} \right)*\gamma*\ln\left( n \right)$	(4)

Where $n$is the number of time points, and $\gamma$is the difficulty of transitioning to a higher state. The total cost of transitioning from one state to another equals the sum of the above two functions. With the cost function established, the optimal state sequence q。can be found. The optimal state sequence is the one that best explains the observed proportions. We use the Viterbi algorithm to find q。

**Reference**

1.	Cao, J., et al., A density-based method for adaptive LDA model selection. Neurocomputing, 2009. 72(7): p. 1775-1781.

2.	Arun, R., et al. On Finding the Natural Number of Topics with Latent Dirichlet Allocation: Some Observations. 2010. Berlin, Heidelberg: Springer Berlin Heidelberg.

3.	Mimno, D., et al., Optimizing semantic coherence in topic models, in Proceedings of the Conference on Empirical Methods in Natural Language Processing. 2011, Association for Computational Linguistics: Edinburgh, United Kingdom. p. 262–272.

4.	AlSumait, L., et al. Topic Significance Ranking of LDA Generative Models. in Machine Learning and Knowledge Discovery in Databases. 2009. Berlin, Heidelberg: Springer Berlin Heidelberg.

5.	Kleinberg, J., Bursty and Hierarchical Structure in Streams. Data Mining and Knowledge Discovery, 2003. 7(4): p. 373-397.
